# Optical analysis of light-emitting electrochemical cells

**DOI:** 10.1038/s41598-019-46860-y

**Published:** 2019-07-18

**Authors:** E. Mattias Lindh, Petter Lundberg, Thomas Lanz, Ludvig Edman

**Affiliations:** The Organic Photonics and Electronics Group, Department of Physics, Umeå University, SE-90187 Umeå, Sweden

**Keywords:** Photonic devices, Organic LEDs, Organic LEDs, Photonic devices

## Abstract

The light-emitting electrochemical cell (LEC) is a contender for emerging applications of light, primarily because it offers low-cost solution fabrication of easily functionalized device architectures. The attractive properties originate in the *in-situ* formation of electrochemically doped transport regions that enclose an emissive intrinsic region, but the understanding of how this intricate doping structure affects the optical performance of the LEC is largely lacking. We combine angle- and doping-dependent measurements and simulations, and demonstrate that the emission zone in our high-performance LEC is centered at ~30% of the active-layer thickness (*d*_al_) from the anode. We further find that the emission intensity and efficiency are undulating with *d*_al_, and establish that the first emission maximum at *d*_al_ ~ 100 nm is largely limited by the lossy coupling of excitons to the doping regions, whereas the most prominent loss channel at the second maximum at *d*_al_ ~ 300 nm is wave-guided modes.

## Introduction

A number of recent studies demonstrate that the light-emitting electrochemical cell (LEC) can be fabricated with low-cost and scalable solution methods^1–6^ on a plethora of functional and novel substrate materials, including fiber^7^, textile^8^, elastomer^9,10^, and paper^11^. As such, the LEC promises to pave the way for an introduction of functionalized devices in a broad range of applications, including medicine^12,13^, security^14^, and communication^15^, where designed light emission can provide unique and important operational advantages.

The enabling factor for these attractive opportunities is the characteristic electrochemical operation during which mobile ions, present through the inclusion of a salt into the organic semiconductor in the active layer, rearrange during the device turn-on. The ions first form electric double layers at the electrode interfaces, which facilitate efficient and balanced hole/electron injection into the organic semiconductor, and then take part in the electrochemical p- and n-type doping of the same organic semiconductor at the anode and cathode, respectively. The doped regions function as high-conductivity electronic charge-transport layers, so that the injected electrons and holes can recombine to excitons, which subsequently can decay under light emission, in the intermediate junction region (note that the *in-situ* formation of mobile dopants makes it cumbersome to define whether the junction is of p-n or p-i-n type, but that we have opted to term it p-n for simplicity reasons)^16–19^.

It is firmly established that the electrochemical doping is highly attractive from an *electrical* point-of-view, since it effectuates efficient and balanced injection of electrons and holes at low voltage. It is also practical from a fabrication viewpoint, since it renders the LEC tolerant to variations of the active-layer thickness (*d*_al_) and the selection of the electrode materials^20–25^. The influence of this doping on the *optical* performance is, however, less understood, although recent reports indicate that an appropriate optical design is paramount if a good LEC performance is to be attained^26–30^.

It is therefore the goal of this study to remedy this shortcoming through a systematic combination of experiments and simulations. Specifically, we aim to establish a good estimate of the *in-situ* formed p-n junction doping structure, to utilize this information for the quantification of the different optical loss channels at different operational conditions, and to provide guidelines for how improved LECs should be designed from an optical perspective. To this end, we have employed LEC devices based on the well-known polymeric semiconductor termed Super Yellow^31–33^, since the evolution of its optical properties with both p- and n-type doping is available^34^, and since high-performance LEC devices based on this semiconductor are common in the scientific literature^30,35–40^.

We find that the effective width of the emissive intrinsic region at steady-state increases in a sublinear manner with the active-layer thickness (*d*_al_), and—somewhat unexpectedly—that the emissive region is centered at ~30% of the active-layer thickness away from the positive anode for all investigated values of *d*_al_. The steady-state emission of these LECs undulates strongly with *d*_al_ because of interference effects, and we report that the optical output of the device operating at the first light-emission maximum (*d*_al_ ~ 100 nm) is mainly limited by the “lossy” coupling of excitons to the doped transport regions. The second-maximum device (*d*_al_ ~ 300 nm) instead primarily suffers from losses to wave-guided modes, although self-absorption within the electrochemically doped regions, doping-induced quenching of excitons, and substrate-guided modes are also significant loss channels. With this information at hand, it is clear that the rational design of efficient thin LEC devices should primarily focus on the suppression of exciton-dopant interactions, whereas thicker and more fault-tolerant LECs in addition should be equipped with optically structured substrates and intermediate layers in order to improve outcoupling, and be designed to suppress the doping-induced self-absorption.

## Results and Discussion

**Figure 1:**
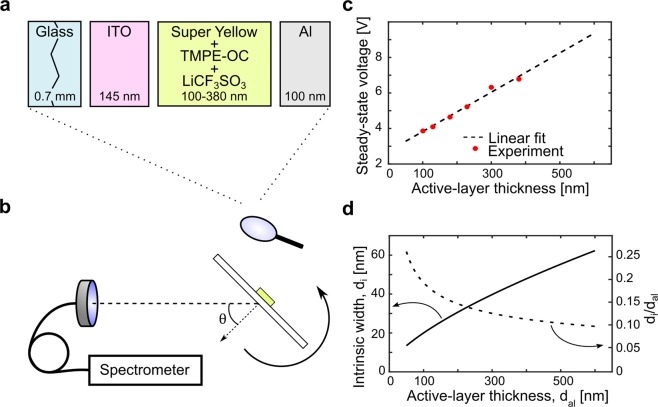
(**a**) Schematic of the LEC device structure, with the thickness of each layer defined. (**b**) The angle-resolved optoelectronic measurement setup with the definition of the viewing angle, θ. (**c**) The measured steady-state voltage as a function of active-layer thickness for the LEC devices driven by a current density of 25 mA cm^−2^, with the dashed black line representing a linear fit of the experimental data. (**d**) The width of the intrinsic region, d_i_, (left y-axis) and the fraction of the active layer occupied by the intrinsic region, d_i_/d_al_, (right y-axis) as a function of the active-layer thickness, as derived with the procedure outlined in the text.

Figure 1(a) presents an exploded view of the device structure, with the glass substrate (thickness: *d*_glass_ = 0.7 mm), the transparent indium-tin-oxide (ITO) anode (*d*_anode_ = 145 nm), the active layer, and the reflective Al cathode (*d*_cathode_ = 100 nm). The active layer comprised the fluorescent conjugated polymer Super Yellow, the salt LiCF_3_SO_3_, and the salt-dissolving and ion-transporting oligomer *n*-octyl carbonate-capped trimethylolpropane ethoxylate (TMPE-OC)^41^. The thickness of the active layer (*d*_al_) was varied between 100 and 380 nm in the experiments, and the overlap between the ITO anode and the Al cathode defined the 2 × 2 mm^2^ emission area. The luminance and the emission spectrum of the LEC devices as a function of the viewing angle (θ) were recorded with the custom-built spectroscopic goniophotometer depicted in Fig. 1(b), with which the emitted light within a small solid angle of 0.007 sr was collected by a collimating lens and delivered by an optical fiber to a calibrated spectrometer, synchronized to the rotation of the device.

The LECs were driven by a constant current density of 25 mA cm^−2^ and observed to reach steady state within 180 min of operation^29^. Figure 1(c) presents the measured steady-state voltage (*V*_ss_) as a function of *d*_al_, and we find that *V*_ss_ increases essentially linearly with *d*_al_. The dashed line is a linear fit of the measured data, which intersects the y-axis at 2.7 V. As this value is close to the energy-gap potential of Super Yellow (*E*_g_/*e* ≈ 2.6 V)^42^, we draw the conclusion that the combined voltage drops over the two electric double layers is well approximated by the energy-gap potential of the electroactive organic semiconductor, as expected for a functional LEC^43^. By making the assumption that the voltage drops over the doped p- and n-type transport regions at steady-state are negligible in comparison to the drop over the undoped intrinsic part of the junction region, in agreement with dedicated potential-profile studies on planar surface cells^18,43,44^, we derive that the voltage drop over the intrinsic region is: *V*_i_ = *V*_ss_ − *E*_g_/*e*.

It is further reasonable that the current density through the intrinsic region at steady state (i.e. at zero ionic current) is space-charge limited, and accordingly obeys the equation: *j*_i_ = *k*_i_ ∙ *V*_i_^2^/*d*_i_^3^ ^45,46^. For bipolar transport, the constant *k*_i_ depends on the recombination rate of electrons and holes^45^, which is unknown for Super Yellow. By setting *d*_i_ = 20 nm at the measured peak efficiency of this system, i.e. at *d*_al_ = 100 nm for which *V*_i_ = 1.3 V (see ref.^29^ and Fig. 1c), we can estimate the value of *k*_i_ to be 1.3 ∙ 10^−21^ Am V^−1^ at *j*_i_ = 25 mA cm^−2^. The motivation for this selection is that the diffusion distance for excitons in organic semiconductors often is measured to be ~10 nm^47–49^, and that the peak efficiency accordingly should be attained at twice this thickness when the diffusion of excitons to the doped regions for quenching by polarons is low, while the positive effects of doping still are largely in place.

This combined experimental and analytical procedure results in the relationship between the effective width of the intrinsic region and the total active-layer thickness displayed in Fig. 1(d). It is interesting that the intrinsic region increases in size with increasing *d*_al_, but that the relative amount of the active layer that is occupied by the intrinsic region, *d*_i_/*d*_al_, actually decreases from 27% at *d*_al_ = 50 nm to 10% at *d*_al_ = 600 nm, as depicted in Fig. 1(d). We note that this general observation of an increasing absolute size, but a lowered occupied fraction, of the intrinsic region with increasing active-layer thickness is in good qualitative agreement with previous studies on LEC systems^18,44,50–52^.

**Figure 2:**
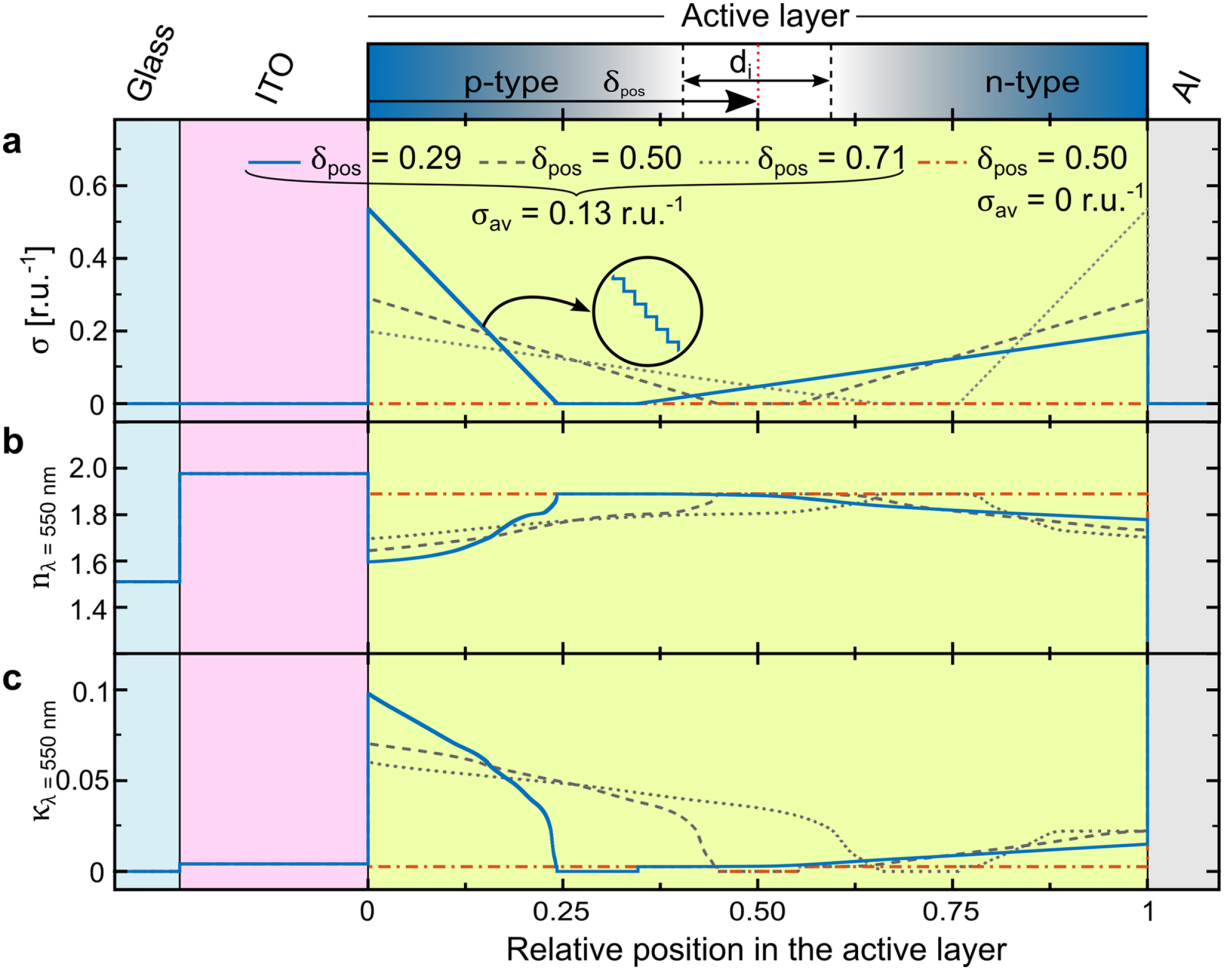
(**a**) The schematic doping structure of an LEC device operating at steady-state, with the width of the intrinsic region, d_i_, its central position in the active layer, δ_pos_, and the position of the electrodes defined. The steady-state doping-concentration profiles for LEC devices with δ_pos_ = 0.29 (solid blue line), δ_pos_ = 0.50 (dashed line), and δ_pos_ = 0.71 (dotted line). The trivial doping profile for a doping-free device (dash-dotted red line) is included as a reference. The corresponding spatial profiles for (**b**) the refractive index and (**c**) the extinction coefficient at λ = 550 nm.

We now shift our attention to the analysis of the doping structure of the p- and n-type regions. First of all, for a balanced LEC device void of side reactions, the total amount of p-type and n-type doping must be equal on the basis of charge conservation and redox balance^53,54^. Moreover, at steady state, all ions will be locked up in doping (and by the electric double layers), so that one can estimate the average doping concentration in the active layer by the initial ion concentration^55,56^. Thus, for the herein investigated devices, the *average* total doping concentration of the entire active layer at steady state, is σ_av_ = 0.13 dopants per Super-Yellow repeat unit (see Methods). The gradient of doping is more difficult to pinpoint, but in agreement with the simulation results outlined in ref.^43^ and for simplicity, we opted to set the doping gradients constant, with the peak concentration at the electrode interface and zero concentration at the edge of the intrinsic region. Figure 2(a) finally defines the center position of the intrinsic region (δ_pos_) as the distance from the ITO-anode/active-layer interface normalized by the active-layer thickness, implying that δ_pos_ = 0.50 corresponds to an intrinsic region in the middle of the active layer and δ_pos_ = 1 to an intrinsic region centered at the active-layer/Al-cathode interface.

Figure 2(a) also presents three different doping structures for devices with the intrinsic region in the middle of the active layer (δ_pos_ = 0.50, dashed line), with an anode-shifted intrinsic region (δ_pos_ = 0.29, solid blue line), and with a cathode-shifted intrinsic region (δ_pos_ = 0.71, dotted line). A direct consequence of the doping balance and the employed constant doping gradients is that a smaller sized doping region features a larger peak doping concentration (at the electrode interface) than a larger sized doping region. We have also included a hypothetical device with zero doping in the active layer (red dash-dotted line) in order to highlight the effects of doping.

Figure 2(b,c) present the corresponding spatial profiles for the refractive index (n) and the extinction coefficient (κ), respectively, of the active layer for the four doping scenarios. The doping-dependent values for n and κ were retrieved and interpolated from ref.^34^, but the highest-concentration values were not directly available and therefore extrapolated with the procedure outlined in the context of Fig. S1. We selected to present the values for n and κ at λ = 550 nm in Fig. 2(b,c), since this is the wavelength of the photoluminescence peak of Super Yellow (Fig. S1b); but in the forthcoming simulations the full wavelength dispersions have been utilized. The general trends (at λ = 550 nm) are that the refractive index decreases in magnitude with doping, while the extinction coefficient increases. We also note that the response of the optical properties of Super Yellow to doping is highly asymmetric, with stronger shifts being observed for p-type doping than n-type doping^34^.

The investigated LEC devices comprise an active layer with a thickness (*d*_al_ = 100–380 nm), comparable to the emission wavelengths (PL_peak_ ~ 550 nm), which is sandwiched between a 145-nm thin semitransparent ITO anode and a reflective Al cathode. As such, the device forms a weak optical microcavity, featuring emission coupled out of the semitransparent ITO anode, which can be strongly influenced by constructive or destructive interference depending on the values for *d*_al_, the viewing angle θ (Fig. 1b), and the position of the intrinsic emission zone δ_pos_ (Fig. 2a).

We have measured and simulated the emission spectrum of our LEC devices as a function of *d*_al_ and θ, and utilize the cavity dependence to establish δ_pos_. For the simulation, we employed the commercial thin-film optics software setfos, which is based on a dipole-emission model that considers the wavelike properties of the emission (see Methods for details). For the simulation, we further used the values for the doping-dependent refractive index and extinction coefficient displayed in Fig. 2(b,c), respectively, while the linear doping gradients were estimated by staircase gradients (see inset in Fig. 2a) comprising 200 constant-concentration sublayers for each region, using the discretization analysis presented in Fig. S2.

A quantitative determination of δ_pos_ is provided by calculating the difference between the measured and simulated spectra, ${{\rm{\Delta }}}_{sim}^{meas}$, as a function of δ_pos._ ${{\rm{\Delta }}}_{sim}^{meas}$ is defined as the sum of the absolute differences between the measured and simulated emission spectra at each measured wavelength and viewing angle divided by the number of comparison points (${N}_{\theta ,\lambda }={N}_{\theta }\cdot {N}_{\lambda }$):

1 $${{\rm{\Delta }}}_{sim}^{meas}=\sum _{\theta }(\sum _{\lambda }|{I}_{\lambda ,\theta }^{meas}({\delta }_{pos})-{I}_{\lambda ,\theta }^{sim}({\delta }_{pos})|)/{N}_{\theta ,\lambda }$$

**Figure 3:**
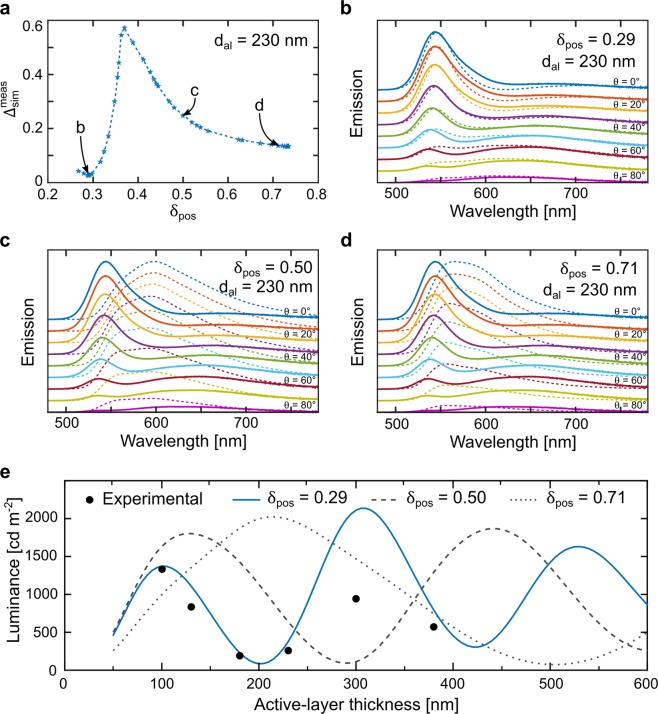
(**a**) The calculated difference between the measured and simulated data as a function of δ_pos_ for the LEC with d_al_ = 230 nm and σ_av_ = 0.13 r.u.^−1^. (**b**–**d**) The measured (solid lines) and simulated (dashed lines) emission spectra for a set of viewing angles ranging from 0° (top trace) to 80° (bottom trace) for the same device. The position of the emissive intrinsic region in the simulation was δ_pos_ = 0.29 (**b**), δ_pos_ = 0.50 (**c**), and δ_pos_ = 0.71 (**d**). The measured and simulated spectra were normalized with respect to the peak intensity at θ = 0°. (**e**) The measured (solid circles) and the simulated forward luminance as a function of active-layer thickness for LECs, with the simulated data being distinguished by the selected position of δ_pos_, as defined in the upper inset.

Figure 3(a) reveals that the best simulation fit of the measured spectra for the LEC with *d*_al_ = 230 nm is obtained with δ_pos_ = 0.29, as the ${{\rm{\Delta }}}_{sim}^{meas}$ function presents a global minimum at this value. A more visually convincing case is perhaps provided by Fig. 3(b–d), which present the measured (solid lines) and the simulated (dashed lines) emission spectra as a function of viewing angle, with the smallest angle (θ = 0°) at the top and the largest angle at the bottom (θ = 80°). The sole distinguishing feature between the simulation data in the three graphs is that the position of the intrinsic emission zone was varied as follows: δ_pos_ = 0.29 (3b), δ_pos_ = 0.50 (3c) and δ_pos_ = 0.71 (3d). It is visually obvious that the best agreement between the measured and simulated spectra is obtained for an off-centered emissive intrinsic region positioned closer to the ITO anode.

We have compared the measured and simulated emission spectra using the same procedure also for the other values of *d*_al_, and a summary of the derived central position for the emissive intrinsic region δ_pos_ and the associated value for ${{\rm{\Delta }}}_{sim}^{meas}$ are presented in Table 1; the corresponding ${{\rm{\Delta }}}_{sim}^{meas}$ vs. δ_pos_ graphs are displayed in Fig. S3(a), while a comparison of the measured and simulated spectra for δ_pos_ = 0.29 is presented in Fig. S4. Importantly, we find that the intrinsic emission zone is positioned closer to the ITO anode for all of the investigated active-layer thickness ranging from 100 to 380 nm, with δ_pos_ being essentially constant at 0.27–0.32. We have also established the position of δ_pos_ for a hypothetical zero doping concentration scenario (as described by the dash-dotted red lines in Fig. 2a–c), and find that the best match between the measured and simulated emission spectra remains for δ_pos_ ≈ 0.3 (see Fig. S3b). This confirms that the finding of an intrinsic emission zone closer to the anode for this specific system is robust with respect to the employed doping concentration. We finally mention that we previously reported a preliminary value of δ_pos_ ≈ 0.6 for this device structure, but that the employed procedure for its derivation was rough and in retrospect inadequate^29^.

**Table 1:** The derived center position of the emissive intrinsic region δ_pos_, and the corresponding difference between the measured and simulated emission spectra, ${{\rm{\Delta }}}_{sim}^{meas}$, for different thicknesses of the active layer.

*d*_al_ [nm]	δ_pos_	${{\rm{\Delta }}}_{{\boldsymbol{sim}}}^{{\boldsymbol{meas}}}$
100	0.29	0.021
130	0.27	0.027
180	0.30	0.045
230	0.29	0.028
300	0.30	0.023
380	0.32	0.025

Figure 3(e) presents the measured (solid black circles) and the simulated forward luminance as a function of *d*_al_. The measured data were derived by spectral integration of the non-normalized emission spectra at θ = 0°. The simulated data were obtained for the three doping-profile scenarios with δ_pos_ = 0.29 (solid blue line), δ_pos_ = 0.50 (dashed grey line), and δ_pos_ = 0.71 (dotted grey line), as presented in Fig. 2(a). It is clear that both the measured and the simulated forward luminance exhibit a periodic dependence on *d*_al_, with the first measured maximum located at ~100 nm and the second measured maximum at ~300 nm. Moreover, it is also obvious that the period between neighboring simulated maxima is strongly dependent on the selected value for δ_pos_, and that the best agreement with experiments is once again attained with δ_pos_ = 0.29. These observations originate in that the LEC, as discussed above, forms a weak optical microcavity, and that the values of *d*_al_ for which constructive interference (emission maximum) and destructive interference (emission minimum) appear are strongly dependent on the value of δ_pos_.

Up to this point, we have simulated the exciton profile as an infinitely thin Gaussian distribution (effectively a delta distribution) that is centered in the intrinsic region, i.e. coinciding with δ_pos_. In order to test the validity of this approach, we have performed a systematic investigation of the effects of broadening the Gaussian distribution on the replication of the measured properties. Figure S5(a) presents the agreement between experiment and simulation for the emission spectra as a function of the width of the exciton profile, and a comparison with Fig. 3(a) and S3 shows that the sensitivity to a variation of this parameter is weak in comparison to that of δ_pos_. Figure S5(b) further reveals that the periodicity of the forward luminance with respect to the active-layer thickness is well captured independent on the exact width of the exciton profile, but that the best quantitative fit of the measured value of the forward luminance for the peak-performing LEC with a thin active layer of 100 nm is obtained with the delta profile. For this reason, and in order to maintain a feasible computational time, we have selected to estimate the exciton distribution with a sharp delta function in the simulations.

We do however wish to mention that the simulation overestimates the forward luminance at larger values for *d*_al_ in Fig. 3(e), and speculate that this could be due to the employment of a too-narrow or too-symmetric exciton profile, the existence of non-flat interfaces or a non-uniform active material^36^, which induces scattering, the existence of non-linear doping profiles, or an underestimation of the extinction coefficient in the heavily doped regions.

We finally utilize the simulation results for an identification and estimation of the “useful” outcoupled light emission and the different loss channels in LEC devices as a function of the active-layer thickness and the doping concentration. We have omitted the constant losses to dark triplets in this specific analysis for clarity, since it amounts to 75% for a singlet emitter such as Super Yellow, independent of *d*_al_ and doping concentration. These dark-triplet losses were, however, accounted for in all of the previously presented simulation results.

**Figure 4:**
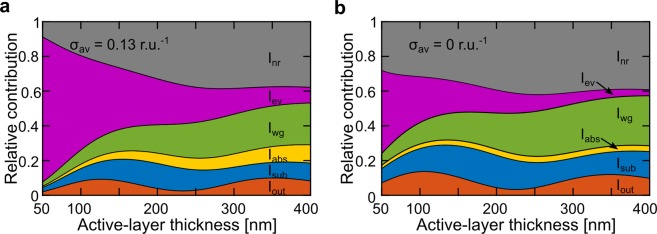
The simulated relative contribution to the total power distribution of (from top to bottom): non-radiative modes (I_nr_, grey area), evanescently coupled modes (I_ev_, purple area), wave-guided modes (I_wg_, green area), linear absorption (I_abs_, yellow area), substrate-bound modes (I_sub_, blue area), and outcoupled modes (I_out_, red area). The simulated data represent (**a**) the LEC during steady-state operation at σ_av_ = 0.13 r.u.^−1^ and (**b**) a hypothetical undoped LEC device with σ_av_ = 0 r.u.^−1^.

Figure 4(a) describes the total power distribution in the LEC during steady-state operation, whereas Fig. 4(b) identifies the corresponding distribution for an identical device with zero doping in the active layer in order to highlight the specific effects of doping. Table 2 presents the data (in percent) for three selected values of *d*_al_, corresponding to the first emission maximum, the first emission minimum, and the second emission maximum, while Fig. S6 displays the relative contribution of each mode as a function of *d*_al_.

**Table 2:** The simulated total power distribution (in %) at the first and second emission maxima and at the first emission minimum for the LEC device at steady state. The values in parenthesis correspond to the power distribution for the hypothetical undoped device.

Emission	*d*_al_ [nm]	*I*_*out*_	*I*_sub_	*I*_abs_	*I*_wg_	*I*_ev_	*I*_nr_
1^st^ max	125 (105)	9 (14)	10 (14)	4 (3)	11 (13)	43 (25)	23 (32)
1^st^ min	230 (225)	3 (3)	13 (17)	6 (4)	19 (24)	23 (11)	36 (41)
2^nd^ max	350 (350)	10 (12)	9 (13)	9 (3)	23 (28)	11 (5)	37 (39)

The total amount of outcoupled light from the device structures (*I*_out_, red area in Fig. 4) features an undulating behavior with *d*_al_, similar to the measured forward-luminance data displayed in Fig. 3(e). This undulating emission intensity can be attributed to the microcavity, and the shift between constructive and destructive interference of the generated light. We further note that the LEC device at steady-state in general features a lower outcoupled emission intensity than the corresponding hypothetical undoped device, and that the absolute outcoupled fraction (the external quantum efficiency) is ~10% at the emission maxima (which corresponds to 2.5% considering losses to the dark triplets).

The amount of optical power lost to modes traveling within the glass substrate (*I*_sub_, blue area in Fig. 4) is also dependent on *d*_al_ in a periodic manner, but it is at this stage unclear as to how the microcavity influences the relative amount of light that is coupled into the (thick) substrate mode. We find that the losses to *I*_sub_ fluctuates between 9 and 13% in the LEC, which is consistently lower than for the hypothetical undoped device. It is important to remember that these modes can be efficiently outcoupled of the device through the relatively straightforward attachment of an appropriate outcoupling structure on top of the substrate^57,58^.

A loss channel that is directly related to the doping concentration and *d*_al_ is the “linear absorption” (*I*_abs_, yellow area in Fig. 4). For the undoped device, *I*_abs_ is consistently low at ~3–4% since the main absorption is effectuated by the electrodes (which are unchanged throughout the experiments). In contrast, for the LEC device *I*_abs_ increases strongly with the active-layer thickness to reach ~10% at *d*_al_ = 400 nm, because of the strong overlap of the emission of Super Yellow with the absorption of the doped transport regions^34,59^.

The losses to waveguiding within the active layer and the ITO anode (*I*_wg_, green area in Fig. 4) increase with *d*_al_, but become lower with doping. The latter decrease is presumably originating in the doping-induced lowering of the refractive index of the active layer within the wavelength range spanned by the Super Yellow emitter (see Fig. S1), which allows some optical modes to leak out from the resulting graded-index active-layer structure depicted in Fig. 2(b). Nevertheless, the losses to *I*_wg_ are significant, particularly at large active-layer thickness with *I*_wg_ being 23% at *d*_al_ = 350 nm, and it thus represents an issue that should be prioritized in the future design of improved LEC devices^27,37^.

The biggest loss channel for the thin LECs is the evanescently coupled modes (*I*_ev_, purple area in Fig. 4), which stem from the near-field absorption and quenching of excitons by the nearby doping regions and by the coupling of excitons to non-emissive surface plasmons at the electrode interfaces. For the LEC at the first emission maximum (*d*_al_ = 125 nm), *I*_ev_ is equal to 43%, but it is notable that it, in contrast to the preceding loss channels, decreases monotonously with increasing *d*_al_. This observation can be rationalized by the increasing average distance between the excitons and the quenching doped transport regions when the intrinsic region grows with increasing active-layer thickness (see Fig. 1d). The coupling of excitons into non-emissive surface plasmons at the electrode surfaces will also decrease with increasing *d*_al_ when the excitons are shifted further away from the electrode interfaces^60^.

The final loss channel is the non-radiative modes (*I*_nr_, grey area in Fig. 4), which to a first approximation is equal to 1 – PLQY_emitter_, i.e. equal to 40% for devices based on the Super Yellow emitter. We do however obtain a lower value for *I*_nr_ at small *d*_al_, which is justified by that the -radiative decay (or the emission) rate of the emitter will increase when the exciton is located close to absorbing media, such as the electrochemically doped transport regions or the semitransparent ITO anode^61^. The doping contribution to this mechanism is manifested in the lower value of *I*_nr_ = 23% at the first emission maximum for the LEC in comparison with the higher value of *I*_nr_ = 32% for the undoped device (Table 2).

## Conclusions

A combination of experiments and simulations is utilized to establish the steady-state doping structure in a high-performance polymer LEC and to identify the different loss channels as a function of active-layer thickness and doping concentration. We find that the center of the emission zone is located at ~30% of the active-layer thickness from the anode, within the investigated range of *d*_al_ of 100 to 380 nm, and that the width of the intrinsic region is increasing in a sublinear manner with *d*_al_. We note that the latter observation is consistent with that the measured width of the intrinsic region in the p-n junction region in open surface cells (with micrometer- or millimeter-sized *d*_al_) commonly is reported to significantly exceed the *d*_al_ of sandwich cells. We observe an undulating variation of the emission intensity and efficiency with *d*_al_, which is well replicated in the simulations, and which is due to a corresponding periodic shift between constructive and destructive interference of the emission envelope within the weak microcavity structure of the device. A particularly important observation relates to the identification and quantification of the steady-state loss channels. We find that the thin LEC operating at the first emission maximum (*d*_al_ ~ 100 nm) is predominately limited by lossy coupling of the excitons in the emission zone to the dopants in the transport regions. The thicker LEC device operating at the second maximum (*d*_al_ ~ 300 nm) suffers from significant losses to doping-induced quenching and self-absorption, but the largest loss channel is waveguide coupling of the emission into internal layers. These findings suggest that a rational design of thin LECs should focus on the development of procedures that limit the coupling between excitons and dopants in the doping regions, whereas a breakthrough in the performance of thicker LECs, which are considered more fit for fault-tolerant low-cost solution processing, will result from the implementation of scalable structuring of the constituent device layers so that the non-desired wave-guiding is suppressed or effectively eliminated.

## Methods

### Device fabrication

The light-emitting and electroactive compound, a phenyl-substituted poly(*para*-phenylenevinylene) copolymer termed Super Yellow (*M*_w_ (average repeat unit) = 338 g mol^−1^, Merck, trade name: PDY-132), was used as received. The ion-transporting compound, an *n*-octyl carbonate-capped trimethylolpropane ethoxylate oligomer (TMPE-OC, *M*_w_ = 918 g mol^−1^), was synthesized according to a procedure described in ref.^41^. The LiCF_3_SO_3_ salt (*M*_w_ = 156 g mol^−1^, Sigma-Aldrich) and TMPE-OC were dried overnight in a vacuum oven at 50 °C before use.

The three active-material constituents were separately dissolved in cyclohexanone in a mass concentration of 10 mg ml^−1^, and thereafter blended in a mass ratio of Super Yellow:TMPE-OC:LiCF_3_SO_3_ = 1:0.2:0.03. This corresponds to a Super Yellow:TMPE-OC:LiCF_3_SO_3_ number ratio of 1:0.074:0.065, with the value for Super Yellow corresponding to the number of repeat units. The blend ink was diluted with additional cyclohexanone to a solute concentration of 7 mg ml^−1^, and thereafter left stirring at 50 °C for ~12 h on a magnetic hot plate.

The active-material ink was spin-coated on top of a carefully cleaned and pre-patterned indium tin-oxide covered glass substrate (*d*_ITO_ = 145 nm, *d*_glass_ = 0.7 mm, Corning Eagle XG glass, Thin Film Devices), and dried at 70 °C for > 6 h on a hotplate. The dry thickness of the active layer (*d*_al_) was controlled by the rotational speed during spin coating (500–1400 rpm, 1000 rpm s^−1^, 60 s), and measured to vary between 100 and 380 nm with contact profilometry (Dektak XT, Bruker). An Al top electrode was deposited on top of the active layer by physical vapor deposition under high vacuum (*p* < 5 ∙ 10^−6^ mbar). The size of the top electrode was defined by a shadow mask, and the overlap of the bottom ITO anode and the top Al cathode established the 2 × 2 mm^2^ emission area. The edges of the glass substrate were blackened with a permanent marker pen to evade detection of wave- and substrate-guided modes. The material processing and the device fabrication were performed in glove boxes under inert N_2_ atmosphere ([O_2_], [H_2_O] < 2 ppm). Devices to be characterized in ambient air were encapsulated by attaching a glass plate on top of the Al electrode with a UV-curable epoxy (Ossilla) using a published procedure^62^.

### Device characterization

The current and voltage were supplied and monitored with a source-measure unit (2400, Keithley). All devices were driven by a constant current density of 25 mA cm^−2^, and the steady-state was obtained within 180 min of operation, as described in ref.^29^. The nonpolarized emission spectrum and luminance was measured with respect to the viewing angle using a custom-built spectroscopic goniophotometer comprising a fiber-optic CCD-array spectrometer (Flame-S, Ocean Optics) and a stepper motor, both controlled by a LabVIEW virtual instrument; see Fig. 1(b). The photoluminescence (PL) spectrum, and the PL quantum yield (PLQY), were measured on thin (~70 nm) films in ambient air using an integrating sphere setup (C9920–02G, Hamamatsu Photonics).

### Simulation

A commercial thin-film optics software was used for the simulations (Setfos 4.6, Emission module, Fluxim AG), which models the excitons as Hertzian dipoles that must be located in a transparent environment. For the specific case of LECs, this mandates the existence of an emissive and transparent region (the intrinsic region) positioned between the absorbing p- and n-type doped transport regions. In consideration of the anticipated unity recombination within the p-n junction, the recombination or exciton-formation current was set equal to the driving current. The orientation of the excitons (the emissive dipoles) was considered isotropic, and the exciton distribution was for computational feasibility centered in the intrinsic region. The exciton-to-photon conversion efficiency was set to 25%, since Super-Yellow is a fluorescent emitter; the losses to dark triplets were for clarity not displayed in Fig. 4, but considered in the remainder of the analysis. The PLQY of the Super-Yellow emitter was 60%. The Purcell effect, i.e. the spontaneous enhancement of an emitter’s emission rate when included into an optical structure, was considered by the “mean wavelength” quenching option in the software. The complex refractive index of the device constituents were available in the literature^34,63,64^, with the exception of the high doping-concentration values for Super Yellow, which were extrapolated from data in ref.^34^ using the procedure discussed in the context of Fig. S1 and Tables S1 and S2. The (0.7 mm) thick glass substrate was considered incoherent in the simulations. The linear doping concentration profiles (Fig. 2a) were implemented into the simulation by dividing the two doped regions into subsets of thinner layers, each with a constant doping concentration. Following the systematic analysis disclosed in Fig. S2, it was established sufficient to divide each of the two doping regions into 200 equally thick sublayers in order to attain a discretization-independent value for the simulated luminance.

The simulations were carried out with the aid of Matlab scripts that generated the necessary simulation input files, organized the execution of the setfos kernel, and collected the results from the generated output files. The fit of δ_pos_ was performed using the built-in minimization routine *fminbnd* in Matlab with an exit tolerance of 10^−3^, and lower and upper bounds of δ_pos_ of 0.265 and 0.735, respectively.

## Supplementary information

Optical analysis of light-emitting electrochemical cells

## References

[1] Liang JJ, Li L, Niu XF, Yu ZB, Pei QB (2013). Fully Solution-Based Fabrication of Flexible Light-Emitting Device at Ambient Conditions. Journal of Physical Chemistry C.

[2] Sandström A, Asadpoordarvish A, Enevold J, Edman L (2014). Spraying Light: Ambient-Air Fabrication of Large-Area Emissive Devices on Complex-Shaped Surfaces. Advanced Materials.

[3] Sandström A, Dam HF, Krebs FC, Edman L (2012). Ambient fabrication of flexible and large-area organic light-emitting devices using slot-die coating. Nature Communications.

[4] Hernandez-Sosa, G. *et al*. The Compromises of Printing Organic Electronics: A Case Study of Gravure-Printed Light-Emitting Electrochemical Cells. *Advanced Materials*, 3235–3240, 10.1002/adma.201305541 (2014). 10.1002/adma.201305541 24616075

[5] Jürgensen N, Zimmermann J, Morfa AJ, Hernandez-Sosa G (2016). Biodegradable Polycaprolactone as Ion Solvating Polymer for Solution-Processed Light-Emitting Electrochemical Cells. Scientific Reports.

[6] Zimmermann, J. *et al*. Ultrathin Fully Printed Light-Emitting Electrochemical Cells with Arbitrary Designs on Biocompatible Substrates. *Adv. Mater. Technol*. **0**, 1800641, 10.1002/admt.201800641.

[7] Zhang ZT (2015). A colour-tunable, weavable fibre-shaped polymer light-emitting electrochemical cell. Nature Photonics.

[8] Lanz T (2016). A light–emission textile device: conformal spray-sintering of a woven fabric electrode. Flexible and Printed Electronics.

[9] Filiatrault HL, Porteous GC, Carmichael RS, Davidson GJE, Carmichael TB (2012). Stretchable Light-Emitting Electrochemical Cells Using an Elastomeric Emissive Material. Advanced Materials.

[10] Yu ZB, Niu XF, Liu ZT, Pei QB (2011). Intrinsically Stretchable Polymer Light-Emitting Devices Using Carbon Nanotube-Polymer Composite Electrodes. Advanced Materials.

[11] Asadpoordarvish A (2015). Light-Emitting Paper. Advanced Functional Materials.

[12] Chaves MED, de Araujo AR, Piancastelli ACC, Pinotti M (2014). Effects of low-power light therapy on wound healing: LASER x LED. An. Brasil. Dermatol..

[13] Takahiko Y, Hajime N, Shigeo H, Toru H, Chihaya A (2017). Near-infrared organic light-emitting diodes for biosensing with high operating stability. Appl. Phys. Express.

[14] Fischer B, Kreissl S, Boeffel C, Wedel A (2012). Multi-layer printing of OLEDs as a tool for the creation of security features. Opt. Express.

[15] Haigh PA (2014). Visible light communications: real time 10 Mb/s link with a low bandwidth polymer light-emitting diode. Opt. Express.

[16] Pei QB, Yu G, Zhang C, Yang Y, Heeger AJ (1995). Polymer Light-Emitting Electrochemical-Cells. Science.

[17] Pei QB, Yang Y, Yu G, Zhang C, Heeger AJ (1996). Polymer light-emitting electrochemical cells: *In situ* formation of a light-emitting p-n junction. Journal of the American Chemical Society.

[18] Matyba P, Maturova K, Kemerink M, Robinson ND, Edman L (2009). The dynamic organic p-n junction. Nature Materials.

[19] Park, J., Shanmugasundaram, K., John, J. C. & Choe, Y. Aggregation Induced Emission Small Molecules for Blue Light-Emitting Electrochemical Cells. *Journal of Photochemistry and Photobiology A: Chemistry*, 10.1016/j.jphotochem.2019.01.011 (2019).

[20] Gao J (2017). Bipolar Electrode Array Embedded in a Polymer Light-Emitting Electrochemical Cell. Acs Applied Materials & Interfaces.

[21] Gao J, Dane J (2003). Planar polymer light-emitting electrochemical cells with extremely large interelectrode spacing. Applied Physics Letters.

[22] Matyba P, Yamaguchi H, Chhowalla M, Robinson ND, Edman L (2011). Flexible and Metal-Free Light-Emitting Electrochemical Cells Based on Graphene and PEDOT-PSS as the Electrode Materials. Acs Nano.

[23] Shin, J. H. *et al*. Light emission at 5 V from a polymer device with a millimeter-sized interelectrode gap. *Applied Physics Letters* **89** (2006).

[24] Sun Q, Yang C, He G, Li Y, Wang H (2003). Effects of electrode modifications on the performance of polymer light-emitting electrochemical cells. Synthetic Metals.

[25] Murto, P. *et al*. High Performance All-Polymer Photodetector Comprising a Donor–Acceptor–Acceptor Structured Indacenodithiophene–Bithieno[3,4-c]Pyrroletetrone Copolymer. *ACS Macro Letters*, 395–400, 10.1021/acsmacrolett.8b00009 (2018). 10.1021/acsmacrolett.8b00009 35619351

[26] Lin, G.-R. *et al*. Non-doped solid-state white light-emitting electrochemical cells employing the microcavity effect. *Physical Chemistry Chemical Physics*, 10.1039/C4CP05380J (2015). 10.1039/c4cp05380j 25679194

[27] Cheng, C.-Y. *et al*. Enhancing device efficiencies of solid-state white light-emitting electrochemical cells by employing waveguide coupling. *Journal of Materials Chemistry C*, 10.1039/C5TC00765H (2015).

[28] van Reenen S, Akatsuka T, Tordera D, Kemerink M, Bolink HJ (2013). Universal Transients in Polymer and Ionic Transition Metal Complex Light-Emitting Electrochemical Cells. Journal of the American Chemical Society.

[29] Lindh, E. M., Lundberg, P., Lanz, T., Mindemark, J. & Edman, L. The Weak Microcavity as an Enabler for Bright and Fault-tolerant Light-emitting Electrochemical Cells. *Scientific Reports* **8**, 10.1038/s41598-018-25287-x (2018). 10.1038/s41598-018-25287-x PMC5934366 29725061

[30] Diethelm, M. *et al*. Optimized Electrolyte Loading and Active Film Thickness for Sandwich Polymer Light-Emitting Electrochemical Cells. *Advanced Optical Materials* **0**, 1801278, 10.1002/adom.201801278.

[31] Gambino S, Bansal AK, Samuel IDW (2013). Photophysical and charge-transporting properties of the copolymer SuperYellow. Organic Electronics.

[32] Jankus V (2013). Energy Upconversion via Triplet Fusion in Super Yellow PPV Films Doped with Palladium Tetraphenyltetrabenzoporphyrin: a Comprehensive Investigation of Exciton Dynamics. Advanced Functional Materials.

[33] Snedden EW, Cury LA, Bourdakos KN, Monkman AP (2010). High photoluminescence quantum yield due to intramolecular energy transfer in the Super Yellow conjugated copolymer. Chem. Phys. Lett..

[34] Lanz T, Lindh EM, Edman L (2017). On the Asymmetric Evolution of the Optical Properties of a Conjugated Polymer during Electrochemical p- and n-type Doping. Journal of Materials Chemistry C.

[35] Noguchi Y, Higeta T, Yonekawa F (2018). Simultaneous Observation of the Electrical and Luminous Characteristics of Light-Emitting Electrochemical Cells by Using a Displacement Current Measurement Technique. Advanced Optical Materials.

[36] Kawecki M (2018). Direct Measurement of Ion Redistribution and Resulting Modification of Chemical Equilibria in Polymer Thin Film Light-Emitting Electrochemical Cells. ACS Applied Materials & Interfaces.

[37] Sato K (2017). Low-Cost, Organic Light-Emitting Electrochemical Cells with Mass-Producible Nanoimprinted Substrates Made Using Roll-to-Roll Methods. Adv. Mater. Technol..

[38] Ni G, Nguyen TD, Vardeny ZV (2011). Study of magneto-electroluminescence and magneto-conductance in polymer light-emitting electrochemical cells. Applied Physics Letters.

[39] Yu Z (2011). Stabilizing the Dynamic p-i-n Junction in Polymer Light-Emitting Electrochemical Cells. The Journal of Physical Chemistry Letters.

[40] Edman L, Pauchard M, Moses D, Heeger AJ (2004). Planar polymer light-emitting device with fast kinetics at a low voltage. Journal of Applied Physics.

[41] Mindemark J (2016). High-Performance Light-Emitting Electrochemical Cells by Electrolyte Design. Chemistry of Materials.

[42] Sandström A, Matyba P, Edman L (2010). Yellow-green light-emitting electrochemical cells with long lifetime and high efficiency. Applied Physics Letters.

[43] van Reenen S (2010). A Unifying Model for the Operation of Light-Emitting Electrochemical Cells. Journal of the American Chemical Society.

[44] Pingree LSC, Rodovsky DB, Coffey DC, Bartholomew GP, Ginger DS (2007). Scanning kelvin probe imaging of the potential profiles in fixed and dynamic planar LECs. Journal of the American Chemical Society.

[45] Pope, M. & Swenberg, C. E. *Electronic processes of organic crystals and polymers*. 2nd edn, (Oxford University Press, 1999).

[46] Lenes M (2011). Operating Modes of Sandwiched Light-Emitting Electrochemical Cells. Advanced Functional Materials.

[47] Kannan B, Castelino K, Majumdar A (2003). Design of Nanostructured Heterojunction Polymer Photovoltaic Devices. Nano Letters.

[48] Tamai Y, Ohkita H, Benten H, Ito S (2015). Exciton Diffusion in Conjugated Polymers: From Fundamental Understanding to Improvement in Photovoltaic Conversion Efficiency. The Journal of Physical Chemistry Letters.

[49] Mikhnenko OV, Blom PWM, Nguyen T-Q (2015). Exciton diffusion in organic semiconductors. Energy & Environmental Science.

[50] AlTal F, Gao J (2016). High resolution scanning optical imaging of a frozen polymer p-n junction. Journal of Applied Physics.

[51] Gao, J. & Dane, J. Imaging the doping and electroluminescence in extremely large planar polymer light-emitting electrochemical cells. *Journal of Applied Physics* **98** (2005).

[52] Hu Y, Gao J (2011). Direct Imaging and Probing of the p−n Junction in a Planar Polymer Light-Emitting Electrochemical Cell. Journal of the American Chemical Society.

[53] Shin JH, Robinson ND, Xiao S, Edman L (2007). Polymer light-emitting electrochemical cells: Doping concentration, emission-zone position, and turn-on time. Advanced Functional Materials.

[54] Fang J, Matyba P, Robinson ND, Edman L (2008). Identifying and alleviating electrochemical side-reactions in light-emitting electrochemical cells. Journal of the American Chemical Society.

[55] Fang JF, Matyba P, Edman L (2009). The Design and Realization of Flexible, Long-Lived Light-Emitting Electrochemical Cells. Advanced Functional Materials.

[56] Fang JF, Yang YL, Edman L (2008). Understanding the operation of light-emitting electrochemical cells. Applied Physics Letters.

[57] Kaihovirta N, Larsen C, Edman L (2014). Improving the Performance of Light-Emitting Electrochemical Cells by Optical Design. ACS Applied Materials & Interfaces.

[58] Jang, Y.-F. *et al*. Enhancing extracted electroluminescence from light-emitting electrochemical cells by employing high-refractive-index substrates. *Organic Electronics*, 10.1016/j.orgel.2017.09.024 (2017).

[59] Kaihovirta N, Asadpoordarvish A, Sandström A, Edman L (2014). Doping-Induced Self-Absorption in Light-Emitting Electrochemical Cells. Acs Photonics.

[60] Penninck, L., Mladenowski, S. & Neyts, K. The effects of planar metallic interfaces on the radiation of nearby electrical dipoles. *Journal of Optics* **12**, 10.1088/2040-8978/12/7/075001 (2010).

[61] van Reenen S, Vitorino MV, Meskers SCJ, Janssen RAJ, Kemerink M (2014). Photoluminescence quenching in films of conjugated polymers by electrochemical doping. Phys. Rev. B.

[62] Asadpoordarvish, A., Sandström, A., Tang, S., Granström, J. & Edman, L. Encapsulating light-emitting electrochemical cells for improved performance. *Applied Physics Letters* **100**, 10.1063/1.4714696 (2012).

[63] Cushman CV (2016). Eagle XG (R) glass, optical constants from 230 to 1690 nm (0.73–5.39 eV) by spectroscopic ellipsometry. Surface Science Spectra.

[64] McPeak KM (2015). Plasmonic Films Can Easily Be Better: Rules and Recipes. Acs Photonics.

