# Supplementary material for: Optical analysis of light-emitting electrochemical cells
Source: Sci Rep. 2019 Jul 18;9:10433. doi: 10.1038/s41598-019-46860-y (PMC6639418; doi:10.1038/s41598-019-46860-y)
Supplement: Supplementary file 1 — Optical analysis of light-emitting electrochemical cells [file 41598_2019_46860_MOESM1_ESM.pdf]

Supplementary information:
Optical analysis of light-emitting electrochemical cells

E. Mattias Lindh, Petter Lundberg, Thomas Lanz, and Ludvig Edman*

The Organic Photonics and Electronics Group, Department of Physics, Umeå University, SE-90187 Umeå, Sweden

*Corresponding author e-mail address: ludvig.edman@umu.se

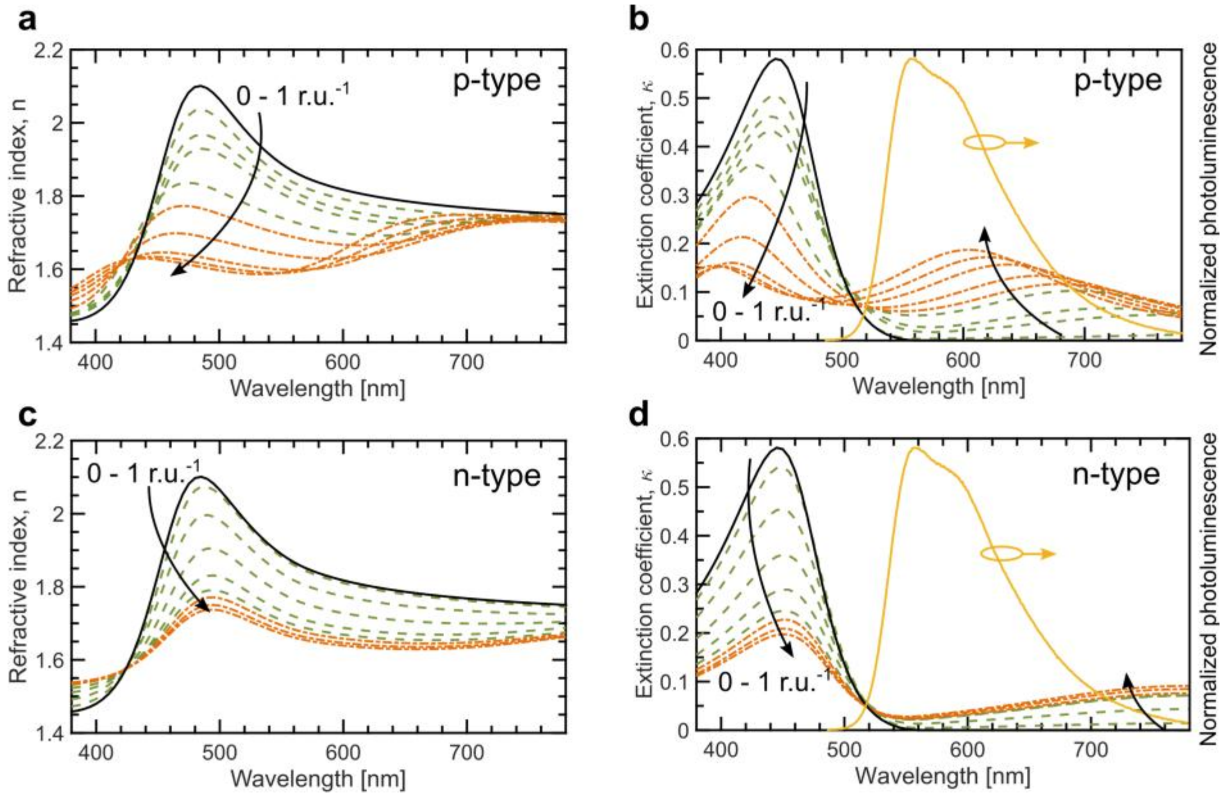

Figure S1. *The refractive index (a, c) and the extinction coefficient (b, d) as a function of wavelength during p-type doping (a, b) and n-type doping (c, d). The arrows indicate increasing doping concentration. The black line indicates pristine undoped Super Yellow, the dashed green lines measured electrochemically doped Super Yellow, and the dash-dotted orange lines extrapolated values for heavier doped Super Yellow, as derived with the procedure described below. The yellow solid lines in (b, d) show the thin-film photoluminescence spectrum of Super Yellow, to visualize its overlap with the extinction coefficient of doped Super Yellow.*

The optical simulations require values for the complex refractive index of Super Yellow at doping concentrations that exceed those available in the scientific literature.[1] For this reason, we have carefully extrapolated the measured values up to 1 dopant per polymer repeat unit. For both p- and n-type electrochemical doping of Super Yellow, the procedure entailed separate extrapolations of the parameters of two Tauc-Lorenz oscillators. The extrapolated values for p- and n-type doping are presented in Table S1 and S2, respectively. Extrapolating the oscillator values rather than the refractive index spectra directly ensures that the Kramers-Kronig relationship between the real and imaginary part is not violated. The real and imaginary part of the complex refractive index presented in Figs. S1 (a-d) were extracted from the oscillator values by using $\varepsilon = (n + i\imath)^2$ and the Planck-Einstein relation $E = hc/\lambda$.

Table S1. *Values of the Tauc-Lorentz oscillator coefficients for p-type electrochemical doping of Super Yellow. The values below 0.2 r.u.$^{-1}$ are derived in Ref. [1], and the values between 0.2 and 1 r.u.$^{-1}$ represent extrapolations from these data.*

σ [r.u.$^{-1}$]	ε(∞)	A_1 [eV]	C_1 [eV]	$E_{0,1}$ [eV]	$E_{g,1}$ [eV]	A_2 [eV]	C_2 [eV]	$E_{0,2}$ [eV]	$E_{g,2}$ [eV]
0	2.5	30.0	0.46	2.65	2.20	0	0.63	1.30	0.68
0.002	2.5	28.0	0.50	2.65	2.20	0	0.63	1.30	0.68
0.005	2.5	27.2	0.50	2.65	2.20	0.17	0.63	1.30	0.68
0.01	2.5	24.5	0.52	2.65	2.18	0.24	0.63	1.40	0.68
0.019	2.5	19.0	0.56	2.67	2.12	0.34	0.63	1.62	0.68
0.039	2.5	16.0	0.58	2.68	2.09	0.40	0.63	1.71	0.68
0.077	2.5	13.7	0.61	2.72	2.05	0.46	0.63	1.73	0.68
0.116	2.5	11.3	0.64	2.76	2.01	0.52	0.63	1.76	0.68
0.155	2.5	8.90	0.67	2.79	1.98	0.58	0.63	1.78	0.68
0.193	2.5	6.60	0.71	2.83	1.94	0.64	0.63	1.80	0.68
0.200	2.5	6.29	0.713	2.83	1.93	0.639	0.63	1.81	0.68
0.300	2.5	3.81	0.756	2.89	1.87	0.701	0.63	1.86	0.68
0.400	2.5	2.80	0.788	2.93	1.83	0.744	0.63	1.90	0.68
0.500	2.5	2.38	0.813	2.97	1.81	0.778	0.63	1.93	0.68
0.600	2.5	2.20	0.834	3.00	1.80	0.806	0.63	1.96	0.68
0.700	2.5	2.13	0.852	3.03	1.79	0.829	0.63	1.98	0.68
0.800	2.5	2.10	0.867	3.05	1.78	0.849	0.63	2.00	0.68
0.900	2.5	2.09	0.881	3.07	1.78	0.867	0.63	2.01	0.68
1.000	2.5	2.09	0.894	3.09	1.78	0.883	0.63	2.03	0.68

Table S2. *Values of the Tauc-Lorentz oscillator coefficients for p-type electrochemical doping of Super Yellow. The values below 0.6 r.u.$^{-1}$ are derived in Ref. [1], and the values between 0.6 and 1 r.u.$^{-1}$ represent extrapolations from these data.*

σ [r.u.$^{-1}$]	ε(∞)	A_1 [eV]	C_1 [eV]	$E_{0,1}$ [eV]	$E_{g,1}$ [eV]	A_2 [eV]	C_2 [eV]	$E_{0,2}$ [eV]	$E_{g,2}$ [eV]
0	2.5	30.0	0.46	2.65	2.2	0	0.80	1.60	0.40
0.001	2.5	30.0	0.46	2.64	2.2	0	0.80	1.60	0.40
0.002	2.5	30.0	0.46	2.65	2.2	0	0.80	1.60	0.40
0.048	2.5	30.0	0.47	2.63	2.2	0	0.80	1.60	0.40
0.097	2.5	26.1	0.49	2.62	2.2	0.07	0.80	1.60	0.40
0.193	2.5	21.8	0.51	2.60	2.2	0.21	0.80	1.60	0.40
0.290	2.5	17.8	0.53	2.59	2.2	0.34	0.80	1.60	0.40
0.386	2.5	16.4	0.55	2.59	2.2	0.34	0.80	1.60	0.40
0.483	2.5	15.2	0.55	2.59	2.2	0.34	0.80	1.60	0.40
0.579	2.5	14.1	0.55	2.59	2.2	0.35	0.80	1.60	0.40
0.600	2.5	13.9	0.552	2.59	2.2	0.356	0.80	1.60	0.40
0.700	2.5	13.2	0.559	2.59	2.2	0.377	0.80	1.60	0.40
0.800	2.5	12.7	0.564	2.59	2.2	0.396	0.80	1.60	0.40
0.900	2.5	12.3	0.570	2.59	2.2	0.413	0.80	1.60	0.40
1.000	2.5	12.0	0.574	2.59	2.2	0.428	0.80	1.60	0.40

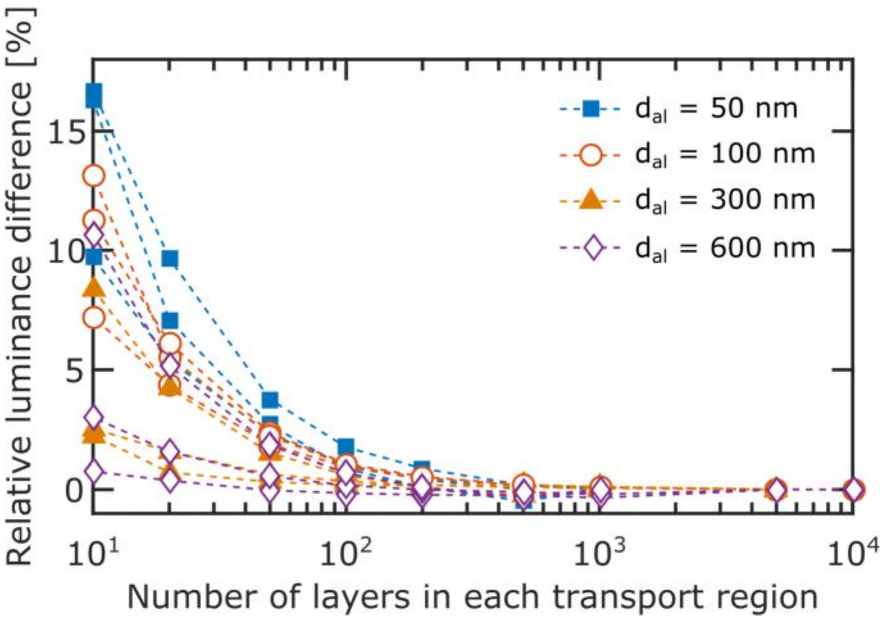

Figure S2. *The relative "error" in the simulated value for the forward luminance as a function of the discretization of the linear doped gradients. The doped layers should ideally be represented by a linear gradient in doping concentration, as depicted in Fig. 2(a), and the "correct" value for the simulated luminance is assigned for a large discretization of* 10^4 *layers for each doped region. The data are collected for four different active-layer thicknesses, as identified in the inset, and for three different center positons for the intrinsic region for each thickness:* δ_{pos} *= 0.265, 0.500, 0.735.*

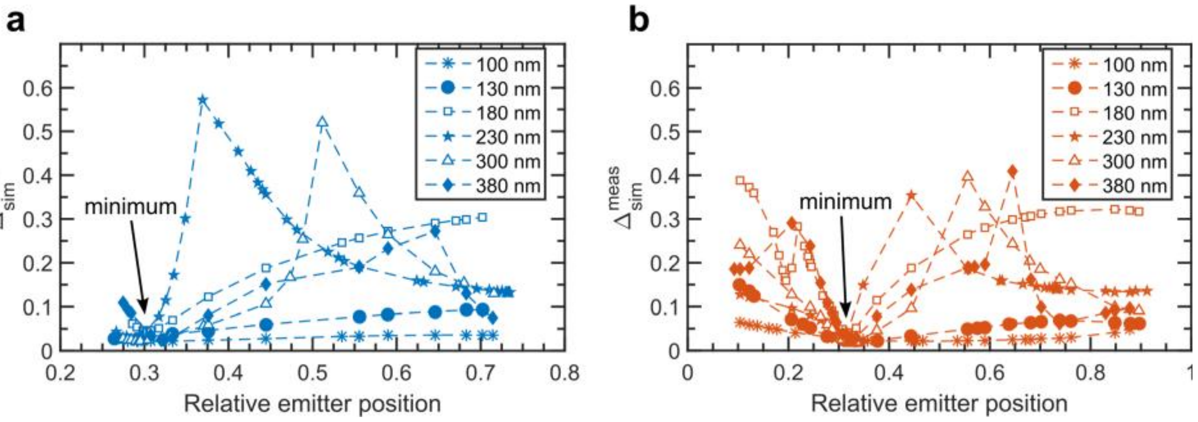

Figure S3. *The mean absolute deviation between the measured and simulated emission spectra,* Δ_{si}^{m} *, as a function of* δ_{pos} *for simulated LEC devices featuring a total average doping concentration of (a) 0.13 dopants per Super Yellow repeat unit and (b) 0 dopants per Super Yellow repeat unit.*

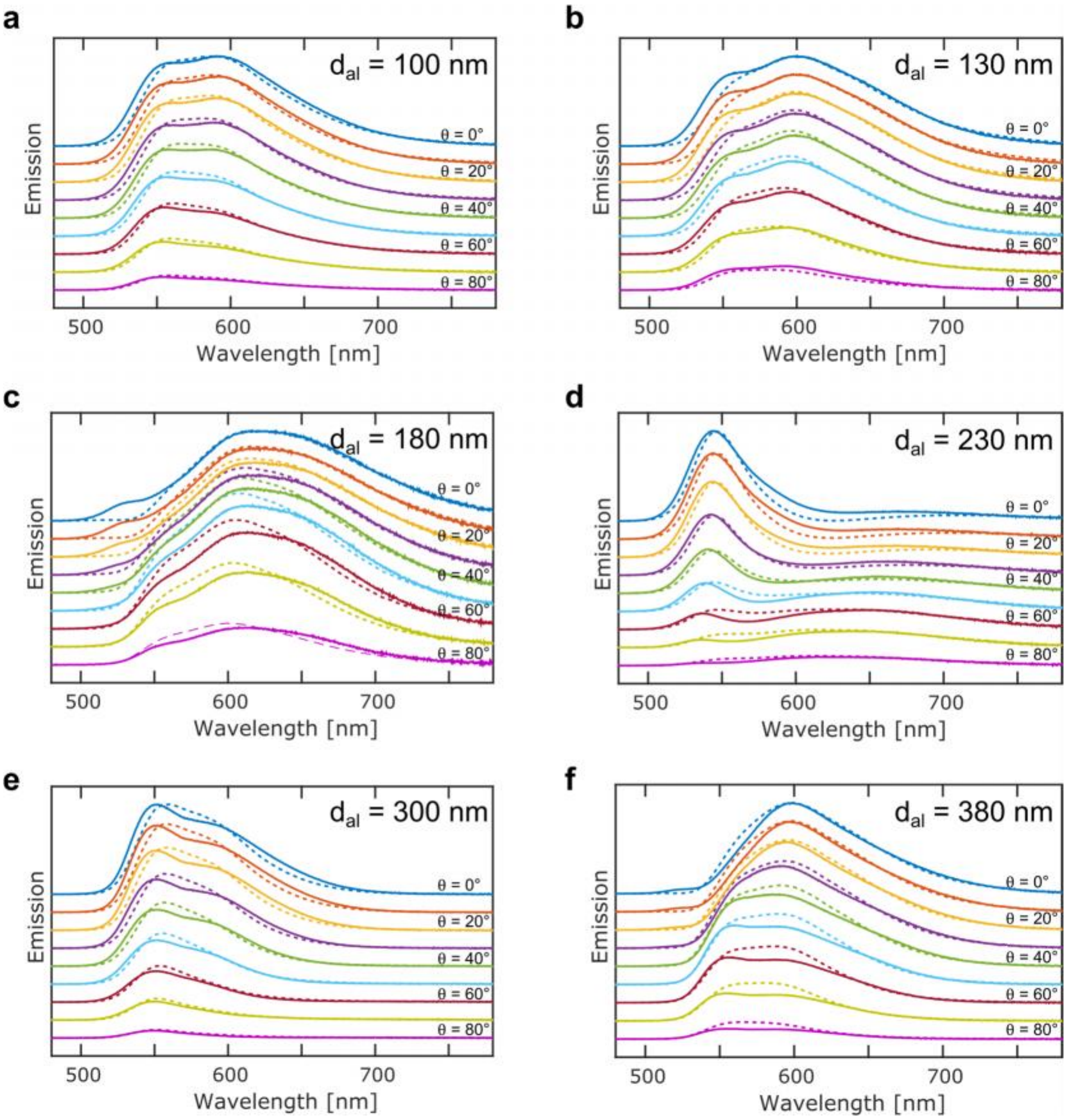

Figure S4. *(a-f) The experimental (solid lines) and the simulated (dashed lines) electroluminescence spectra for* d_{al} *ranging from 100 nm to 380 nm, as specified in the insets. The value for* δ_{pos} *was kept constant at 0.29 in the simulations. The viewing angle ¯ ranged from 0î (top trace) to 80î (bottom trace).*

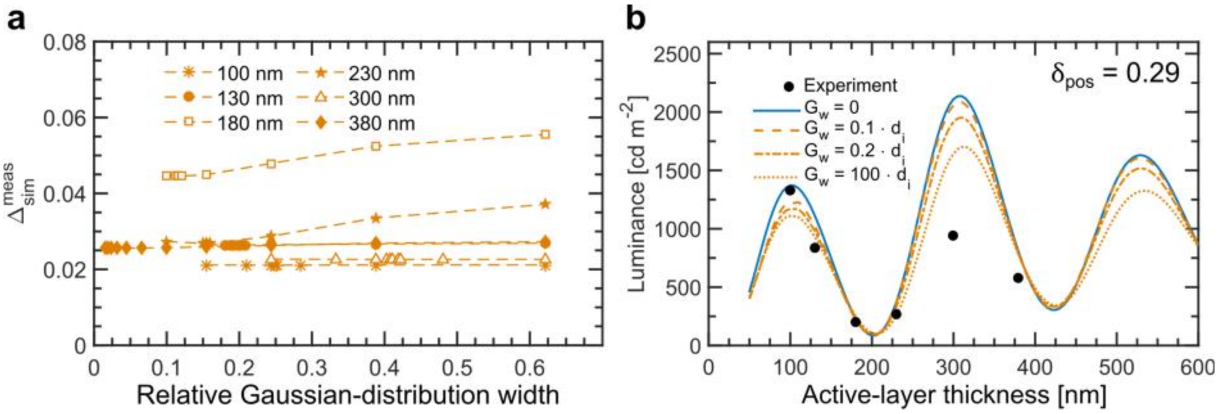

Figure S5. *(a) The mean absolute deviation between the measured and simulated emission spectra,* Δ_{si}^{m} *, as a function of the characteristic width of the Gaussian distribution of the excitons (defined as a multiple of* d_i*). The thickness of the active layer is detailed in the inset. (b) The simulated forward luminance as a function of the active-layer thickness, for different values for the width of the Gaussian distribution of the excitons as specified in the inset. The LEC devices were simulated with* δ_{pos} *= 0.29 and a total average doping concentration of 0.13 dopants per Super Yellow repeat unit. Note that* G_w *= 100* d_i *corresponds to the specific case of a constant flat exciton profile within the intrinsic region.*

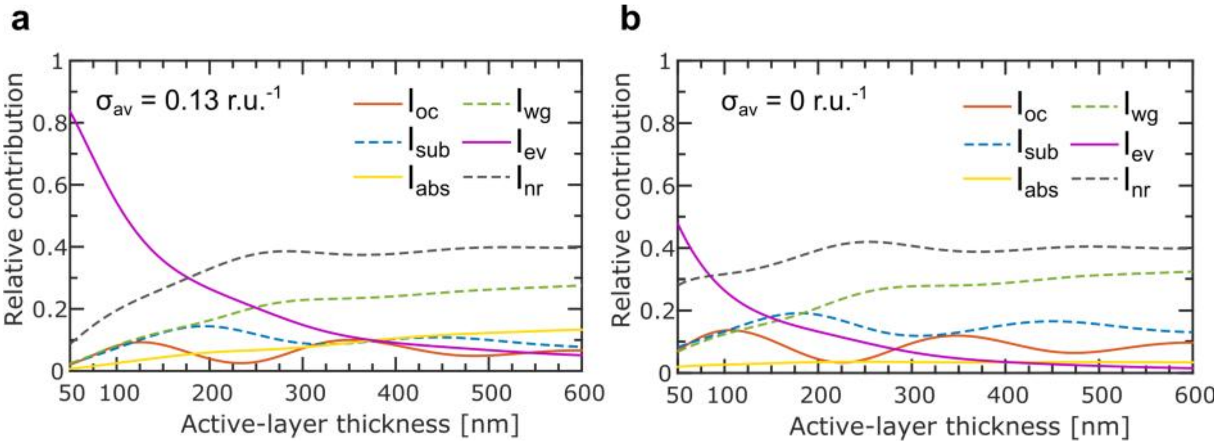

Figure S6. *The simulated relative contribution of the different modes, as identified in the insets, to the total power distribution as a function of active-layer thickness for (a) the LEC during steady-state operation and (b) a hypothetical undoped LEC device.*

References

1 Lanz, T., Lindh, E. M. & Edman, L. On the Asymmetric Evolution of the Optical Properties of a Conjugated Polymer during Electrochemical p- and n-type Doping. *Journal of Materials Chemistry C* **5**, 4706-4715, doi:10.1039/C7TC01022B (2017).
